# Effects of globalization, foreign direct investment and economic growth on renewable electricity consumption

**DOI:** 10.1016/j.heliyon.2023.e14635

**Published:** 2023-03-16

**Authors:** Gulzara Tariq, Huaping Sun, Unai Fernandez-Gamiz, Sofia Mansoor, Amjad Ali Pasha, Sajjad Ali, Muhammad Sohail Khan

**Affiliations:** a School of Finance & Economics, Jiangsu University, Zhenjiang 212013, Jiangsu, China; b School of Economics and Management, Xinjiang University, Urumqi 830046, Xinjiang, China; c Nuclear Engineering and Fluid Mechanics Department, University of the Basque Country UPV/EHU, Nieves Cano 12, 01006 Vitoria-Gasteiz, Spain; d University of Peshawar, Peshawar, Pakistan; e Faculty of Engineering, Aerospace Engineering Department, King Abdulaziz University, Jeddah 21589, Saudi Arabia; f School of Management, Jiangsu University, Zhenjiang 212013, Jiangsu, China; g School of Mathematical Sciences, Jiangsu University, Zhenjiang 212013, Jiangsu, China

**Keywords:** Globalization, Economic growth, Trade openness, Foreign direct investment, Renewable electricity consumption, Belt and road initiative

## Abstract

Renewable energy has been seen as a viable solution to the problems of environmental degradation and the energy crisis. This study examines the long – and short–run linkages between economic globalization, foreign direct investment (FDI), economic growth, and renewable electricity consumption in China’s Belt and Road Initiative (BRI) countries. Therefore, this study uses the Pooled Mean Group (PMG) autoregressive distributed lag (ARDL) technique to measure the relationship between constructs based on data collected from 2000 to 2020. The overall results show the collaborative integration of Belt and Road (BRI) countries in terms of globalization, economic growth, and renewable electricity utilization. The results show that there is a long-term positive relationship between FDI and renewable electricity consumption, but a negative relationship in the short term. Furthermore, economic growth is positively correlated with renewable electricity consumption in the long run and negatively correlated in the short run. This study suggests that the governments of BRI countries should encourage globalization by improving technology and knowledge related to renewable electricity consumption in all areas.

## 1 Introduction

Preservation of energy could be the key to economic development [1]. China’s share in global consumption of energy is 2.9% and 6.1% per year since 2016–2017 and 2000–2017, respectively [2]. The Chinese economy has consistantly ranked higher than the US since 2009 in terms of energy consumption. Quantitatively, China consumed 871 Mtoe in 1990 and 3105 Mtoe in 2017 EIA [3]. While, recovering from the COVID-19 crisis, China's electric power consumption rose by 3.1% and held 29% of the world's electric power consumption in 2020 EnerData [4]. In 2018, nearly 60% of China's coal use was accounted for by the electricity sector, with the balance coming from industry EIA [5]. China's average daily electricity consumption rose from 17.1 to 205.1 (100 million kW-h) from 1990 to 2019 [6]. Moreover, such an increase in energy consumption transmuted China to an energy importer from an energy exporter. Therefore, an affordable and stable energy supply is crucial for economic growth and national security [[7], [8], [9], [10]], such an increase in foreign energy supply has given China a perilous position [[11], [12], [13]].

China began to open its economy and sign several regional trade treaties in the early 1980s. In the meantime, on December 11, 2001 association of China with the World Trade Organization (WTO) began, which underwent another wave of trade, thereby increasing the collective stock from 1085.29 billion to 203.14 billion dollars until 2014. China took the initiative towards BRI (belt and road initiative) during their visit to Kazakhstan and Indonesia in 2013. The primary focus of this initiative was to invest in education, railways, infrastructural investment, the power grid, automobiles, iron, real estate, and highways. At present, 68 countries are contributing to BRI. It was established for the strong relationship between countries, financial development, and excellent connectivity among different countries. Scholars nowadays endeavor to study the link amongst economic growth and energy usage [14,15]. In recent studies, the focus of academics changed from energy use to electricity usage, as obtaining and measuring data is more manageable than energy consumption [16]. Energy consumption analysis is more precisely revealed by electricity consumption analysis [17]. Previously, it was reported that trade openness and FDI create jobs, increase output, and increase real wage growth and technology spillovers [[18], [19], [20], [21]]. In addition, it is easy to state that globalization has been the main factor in modernizing the economy in recent decades. Without a consistent supply of energy, globalization is impossible [22,23].

A long-run asymmetric link between FDI, trade, and energy usage was also accessed in previous studies [24]. Previous studies reported that FDI upturns energy consumption in host economies [[25], [26], [27]]. Renewable energy use, FDI, and trade openness all contribute to economic development [28]. However, the long-term relationship between globalization, economic growth, and renewable electricity has not been well established by previous scholars [[29], [30], [31], [32]]. Therefore, this study seeks to determine the relationship between globalization, economic growth, FDI, and renewable electricity consumption, not only in the short run but also in the long term. This will help decision-makers have a strong understanding of core relationships and facilitate long – and short–term strategic decision-making.

Moreover, this study has made numerous contributions to the literature of energy/electricity economics: (i) this study adds to the current body of knowledge by investigating the dynamic link between renewable and non-renewable electricity usage, where electricity from fossil fuels is considered non-renewable electricity consumption. Foreign direct investment and economic growth potential are also evaluated in the energy demand function to avoid the specification problem. (ii) The second-generation unit root test was used to investigate variable integration (iii) The Pedroni and Kao co-integration approach was applied to examine co-integrating between renewable electricity consumption and its determinants. (iv) PMG ARDL model was applied to examine the impacts of globalization and economic growth on renewable electricity usage; BRI countries were also evaluated according to their regions. (v) To check the causal association among renewable electricity consumption and its factors, the rolling panel causality test is employed.

This study will assist policymakers in developing inclusive energy policies to ensure long-term development. Overall, the results show the cointegration among renewable electricity consumption, trade openness, economic development, and FDI.

The study is structured as follows: Section 2 evaluates the previous work concerning numerous phases of renewable electricity consumption. Part 3 defines the data, methodology, and model. Part 4 explains the findings, and Part 5 discusses the conclusion and policy implications.

## 2 Relevant literature

Many existing works on the link between FDI, economic growth, trade openness, and energy consumption neglected the potential connection among renewable electricity usage, economic growth, trade openness, and FDI in BRI economies. Thus, this negligence may produce misleading results for different countries. Before establishing prior anticipations on these variables, this study discusses the bilateral relation between FDI and renewable electricity consumption, as well as the connection among economic growth and renewable electricity consumption and trade and renewable power consumption. The majority of the existing literature examines the causal relationship between energy/electricity consumption and economic growth using annual data [[33], [34], [35], [36], [37], [38], [39], [40]]. Using a 24-year annual dataset, Acheampong [41] clarified that economic expansion has no influence on global and regional energy consumption.

Apergis et al. [42] focused on a specific geographic region; Eurasia covered the period of 1992–2007 and showed the reaction theory held for renewable power consumption. Apergis and Payne studied Central America and clarified the long-term and short-term feedback and growth consequences of renewable energy. By employing 35 years of panel data, Wolde-Rufael and Yemane discovered the connection among economic development and electricity depletion [43]. Many scholars attempted to explore the connection among economic development and energy/electricity ingesting at the monthly [[44], [45], [46]] or quarterly [17,[47], [48], [49]]. Huang et al. [50] selected 82 countries from 1972 to 2002 by four income groups and reported no confirmation for the growth hypothesis. Omri [51] acquired data from 65 countries from 1990 to 2011 using per capita income and confirmed growth and feedback theory in low-income and middle-income states.

Phrakhruopatnontakitti et al. [52] sustained the existence of two-way connections in Malaysia. Nugraha et al. [53] and Farabi [54] for Indonesia supported a neutral cause among energy consumption and economic development. From 1980 to 2012, Hassine et al. [55] investigated the causality between real GDP, financial development, trade, and renewable energy ingesting in Gulf countries; renewable energy depletion increases economic development in Gulf countries. Cetin et al. [56] used the VECM Granger causality approach to investigate the causality between trade openness, economic development, and energy usage, for upper-middle-income nations from 1971 to 2014. Tariq et al. [57,58] found positive long-run associations among trade openness, energy consumption, and economic development. Sun et al. [59] found a positive association among globalization and environmental pollution in SAARC countries. In Pakistan and India, Tariq et al. [60] examined associations among FDI, economic growth, and trade openness and found positive effects on the environment.

Alam et al. [61] revealed that the long-term usage of renewable energy in OECD nations is highly influenced by economic development, trade openness, and technical advancement. According to the findings of Naimoğlu and Mustafa [62] the long-term coefficient estimation shows that economic development and technical advancement are crucial factors in Turkey's growing usage of renewable energy. Azam et al. [63] used FMOLS and elaborated that economic growth was boosted by 0.095% and 0.017%, respectively, for every 1% increase in the use of renewable and non-renewable electricity. Shahbaz et al. [64] indicated the existence of a long-term connection between the use of renewable energy and economic expansion. Additionally, they noticed that consumption of renewable energy, together with non-renewable energy, labor, and capital, has a favorable influence on economic growth in 38 countries that consume renewable energy.

Ma et al. [65], by regulating significant regional heterogeneity, used provincial data from 1995 to 2004 and concluded the intensity of energy rises because of the use of energy-intensive equipment in the export sector. Even though, Hübler [66] explored the positive correlation among energy production and imports. Though, Shahbaz et al. [67] determined the feedback effect among trade and energy use. Hence, Herrerias et al. [68] used a cross-sectioned time series of provincial data from 1985 to 2008 and explored the inverse association between energy intensity and imports.

Efficient resource allocation in financial markets boosts growth and increases private energy consumption [67,[69], [70], [71]]. Although, affordable prices in the financial markets improve the purchase of consumer durables, ultimately increasing the energy demand [64,72,73]. Hence, the lower the cost of borrowing in the financial market supports households and businesses to get energy preserving technologies, which in the long run decreases the energy demand [74,75].

Smyth et al. [76] reported a significant lack of research as described above in that they all ignored the impacts of renewable energy on FDI and the energy nexus. Paramati et al. [77] investigated the panel data of 20 developing economies during 1991–2012, explored the effects of FDI upon clean energy, and explained the positive long-term association among FDI and renewable energy. Doytch et al. [78] used 74 countries from 1985 to 2012 and demonstrated the positive correlation between renewable energy and FDI. Furthermore, they segregated their sample based on capital income and concluded that FDI in the manufacturing sector reduces renewable energy demand while raising it in financial sector. Salim et al. [75] proposed an unfavorable association among energy and FDI in the short term and a positive association in the long term.

There is currently no research focusing on globalization, economic growth, and renewable electricity usage in BRI countries. Despite globalization's important role in BRI economies, it is critical to investigate the long-run and short-run dynamics of trade openness, economic growth, and renewable electricity use from 2000 to 2020. Therefore, the purpose of this research is to generate the objectives and knowledge necessary to give policy implications to the governments of BRI economies.

## 3 Methodology and estimation methods

### 3.1 Model

Prior research found numerous links between power use, trade openness, and economic development. Smyth et al. [76] explained that the variation might be due to industrial characteristics, economic approaches, variable selection, model specification, and time period selection. To improve these distresses, the following model has been developed:

(1) REC=f(FDI,TO,GDP)

Where REC is renewable electricity consumption, FDI is the flow of foreign direct investment, TO is trade openness used as a proxy forglobalization, and GDP is the real income per capita used as a control variable. The following equation was obtained after applying the linear transformation to empirical investigations on Eq. (1):

(2) $$REC_{it}=\beta_{1}+\beta_{2}FDI_{it}+\beta_{3}TO_{it}+\beta_{4}GDP_{it}+\varepsilon_{it}$$

where t denotes time and $\mu_{t}$ represents Gaussian errors. Eq. 2 β's denotes long-run elasticity. This description also measures the association among electricity consumption and economic growth where technology improvements arise due to increased FDI and trade openness [67].

In our framework, trade openness was the second determinant of electricity consumption. Previous literature shows that FDI and trade openness are other useful determinants for renewable electricity consumption, but economic growth and trade openness increase renewable electricity consumption, so $\beta_{2}$ is positive in Eq. (2).

The increase in exports increases renewable electricity consumption to meet foreign requirements, directly influencing trade openness. Higher exports require extra raw materials and more exporting goods; which increases renewable electricity consumption. This expects the sign $\beta_{3}$ to be positive in Eq. (2), reflecting the positive association among electricity and trade openness. The purpose of this study is to look into the relationship between economic growth, FDI, trade openness, and electricity usage. The literature reveals that the nexus between growth and energy highly depends upon economic development [57]. The positive $\beta_{4}$ in Eq. (2) denotes the positive correlation between economic growth and renewable electricity usage. Currently, no reported work shows the exact sign of $\beta_{3}$ and $\beta_{4}$ in BRI. Therefore, the major goals of this study is to reduce the uncertainty associated with electricity use and increase trade openness.

### 3.2 Data

**Fig. 1:**
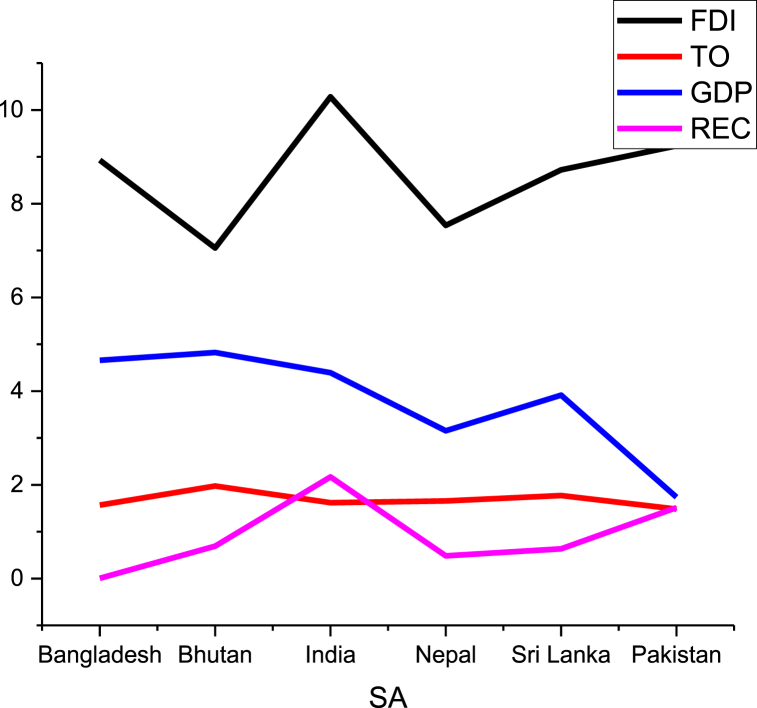
Data distribution of SA countries.

**Fig. 2:**
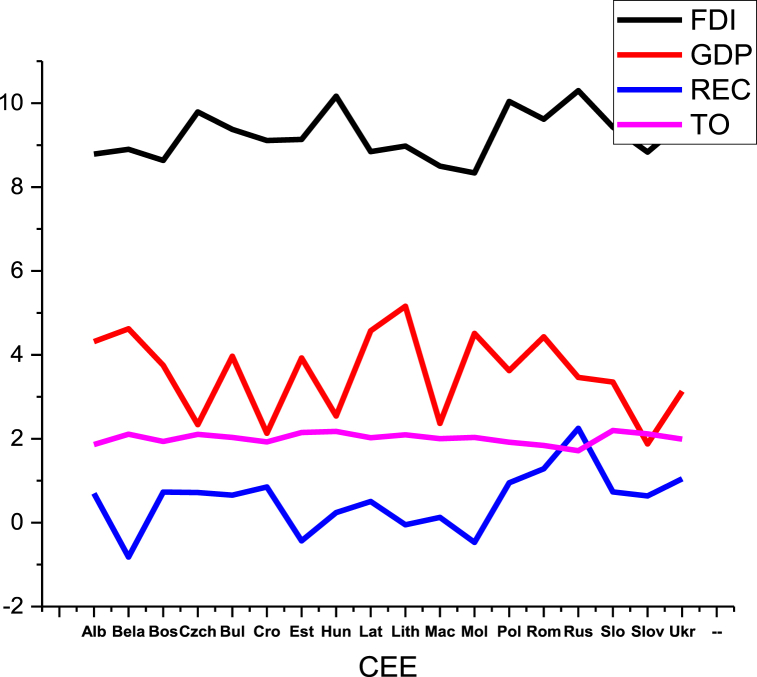
Data distribution of CEE countries.

**Fig. 3:**
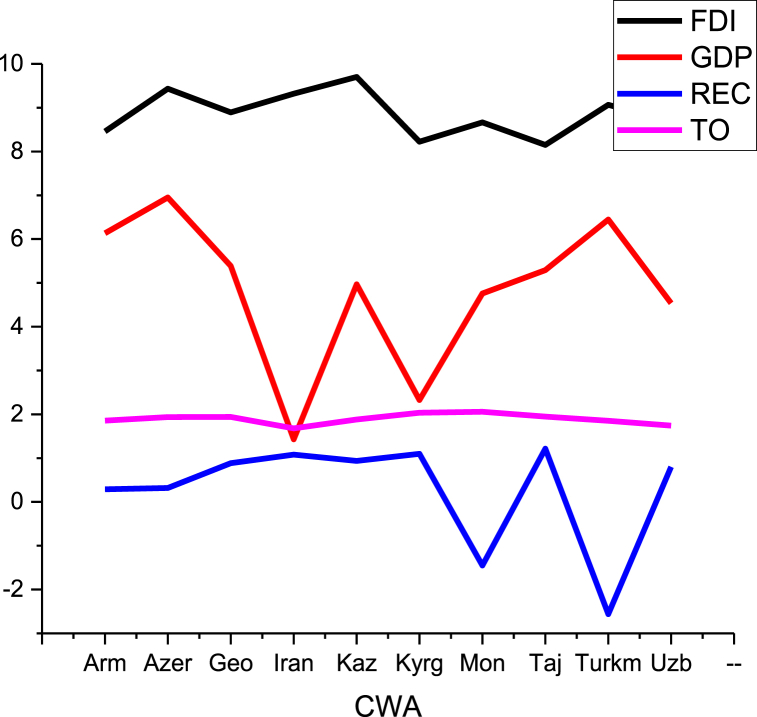
Data distribution of CWA countries.

**Fig. 4:**
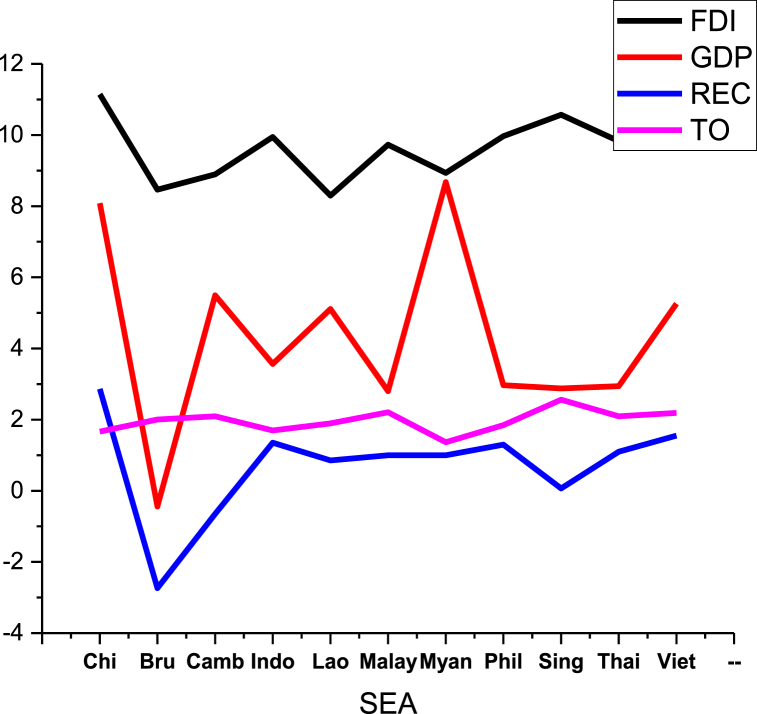
Data distribution of SEA countries.

**Fig. 5:**
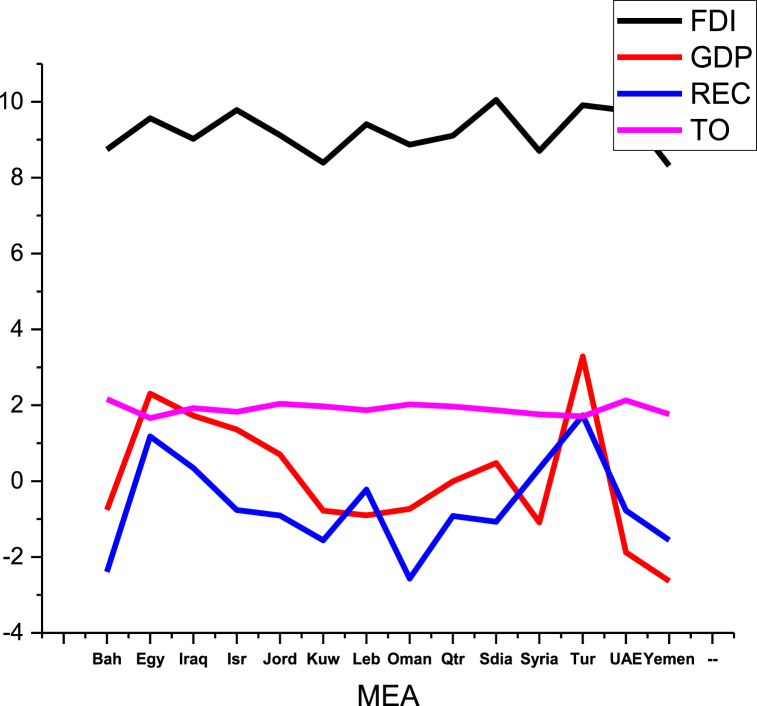
Data distribution of MEA countries.

This research covers the annual observations of BRI from 2000 to 2020 due to the availability of data. To further find out the association between different sub-panels of countries, BRI is divided according to the geographical region named as, the Middle East and Africa (MEA), Central and Western Asia (CWA), South Asia (SA), Central and Eastern Europe (CEE), South East Asia (SEA). Data for trade, economic growth per capita, and FDI are sourced from the World Development Indicators [76]. Trade (as a percentage of GDP) is used as a proxy for trade openness or globalisation, whereas GDP per capita growth annual percentage is used as a proxy for economic growth, FDI is net inflows of foreign direct investment (BoP, current US$). Data related to renewable electricity consumption (REC) is extracted from Our World in Data [79]. All the data is transformed into a natural log. Fig. 1, Fig. 2, Fig. 3, Fig. 4, Fig. 5 depict data trends according to region.

### 3.3 Methodology

This study began with the baseline model and tested the cross-sectional dependency of variables using the Pesaran CD, Pesaran scaled LM, and Breusch-Pagan LM tests. The next step is to investigate variable stationarity in panel data in the case of cross-sectional dependency. According to Kasman et al. [80], each panel unit root test has strengths and weaknesses. Four unit root trials, namely Levin Lin and Chu [81], Breitun J. [82], lm Pesaran and Shin [83], and Phillips-Perron (PP) tests, are applied in this study for increasing robustness. The panel unit root test developed by Levin Lin and Chu [79] is an extension of the augmented Dickey-Fuller test, which is given as

(3) $${\Delta B}_{\text{it}}{=\delta }_{\text{it}}\psi_{i}{+\text{gB}}_{\text{it}-1}+\sum_{k=1}^{\text{mi}}\delta \text{ik}B_{i,t-k}{+\omega }_{\text{it}}$$

Where g represents autoregressive coefficients, $\delta_{\text{it}}$ indicated individual deterministic variables, m is lag order, and ω representing error term.

It is assumed in the Levin Lin and Chu [81] test that g remain constant across countries. Levin Lin and Chu [81] the test is prolonged from lm Pesaran and Shin [83] test, which lets g to vary among countries. Breitun J [82]. is a test that corrects for bias produced by the LLC [81] and IPS [83] tests, as well as gave following equation:

(4) $$B_{it}=\varphi_{it}+\sum_{j=1}^{g+1}\beta_{ij}a_{it-t}+\varepsilon_{it}$$

According to (Hlouskova and Narayan [84,85]), the LPS [82] test has some limitations plus advantages too. The best benefit is that it has the highest power and the smallest sample size biases, whereas the limitation is that the autoregressive coefficient remains the same across countries.

Pedroni [86,87] developed panel and group co-integration tests. Panel rho-Statistics, Panel v-Statistics, Panel pp-Statistics, and Panel ADF-Statistics are all within dimension approaches used by the panel test. It also contains between dimension approaches: Group ADF-Statistics, Group PP-Statistics, and Group rho-Statistics Pedroni's [84,85] cointegration assessment assumes that H0: No co-integration among variablesThese all seven tests, which are asymptotically dispersed as regular standard, are defined as the expected residuals from the long-run model shown below.

(5) $$B_{\text{it}}=\varphi_{i}+\lambda_{i}+\sum_{k=1}^{n}\beta_{\text{ik}}A_{\text{kit}}+\varepsilon_{\text{it}}$$

where A and B are planned to be incorporated into order one levels.

In Eq. (6) the projected residuals are recorded.

(6) $$\varepsilon_{\text{it}}=g_{i}\varepsilon_{\text{it}-1}+\mu_{it}$$

The maximum likelihood-based panel co-integration statistics will be compared to three between-dimension and four within-dimension statistics in this study.

Pedroni's (1999, 2004) study of cointegration system for panel data is stated in Eq. (7) below:

(7) $$B_{\text{it}}=\varphi_{i}+\beta A_{it}+\varepsilon_{it}$$

Kao [88] proposes another co-integration test to estimate the homogeneous co-integration association. Kao [86] proposes two tests for the null hypothesis of no co-integration: the Dickey-Fuller type and the Augmented Dickey-Fuller tests.

Following the establishment of panel co-integration, this research looks at both the short and long run connections among the variables using the PMG ARDL approach. This approach was adopted to allow the mixed order of integrated variables used under the unified framework. Optimal lag length selection also alleviates spurious regression. This broad-based strategy establishes a long-term link between variables. In addition, the structure of lag provides consistent findings and mitigates the issue of serial correlation in the presence of endogenous regressors. It also gives exceptionally consistent long-run and short-run values. The PMG AR distribution lag represents Eq. (2) as follows:

(8) $$lREC_{it}=\delta_{1}lTO_{it}+\delta_{2}lTO_{i,t-1}+\delta_{3}lGDP_{it}+\delta_{4}lGDP_{i,t-1}+\delta_{5}lFDI_{it}+\delta_{6}lFDI_{i,t-1}+\varphi lREC_{i,t-1}+\mu_{i}+\upsilon_{i}$$

The error correction term is provided as:

(9) $$\Delta REC_{it}=\theta_{i}\left(lREC_{i,t-1}-\alpha_{0}-\beta_{1i}lTO_{it}-\beta_{2i}lGDP_{it}-\beta_{3i}lFDI_{it}\right)+\delta_{1}\Delta lTO_{it}+\delta_{2}\Delta lGDP_{it}+\delta_{1}\Delta lFDI_{it}+\sigma_{i}$$

In Eq. (9), an error correction term is denoted by θ, Δ symbolized the first difference operator, the log of variables is signified by l, values of long run are shown by β, short run values are rpresented by δ, ε represented the error term, and t indicated time.

At last, this study outperforms Dumitrescu-Hurlin (DH) panel causality test [89]. This test, for example, includes cross-sectional dependency, and the time and size of the cross-section relative to each other are unimportant. This test presents two separate distributions: asymptotic and semi-asymptotic. When T is greater than N, the asymptotic distribution is used; when N is greater than T, the semi-asymptotic distribution is used. In panel data, the following model finds DH causality:

(10) $$Z_{it}=\alpha_{i}+\sum_{j=1}^{J}\tau_{ii}^{J}Z_{i,t-J}+\sum_{j=1}^{J}\beta_{ii}^{J}X_{i,t-J}+\varepsilon_{i,t}$$

Where $X_{i,t}$ and $Z_{i,t}$ are the observations of two stationary variables for individual I in period t, j is the lag length, $\tau_{i}^{J}$ is the autoregressive parameter, and $\beta_{i}^{J}$ is the regression coefficient that changes across groups. With a balanced panel, it is believed that lag order J is the same for all people. This is a fixed-type test that produces a fixed coefficient model. It enables heterogeneity while preserving normal distribution.

## 4 Empirical results and discussion

The mean, maximum, and standard deviation of trade openness, renewable power consumption, economic growth, and FDI are shown in Table 1.

**Table 1:** Descriptive statistics.

Variables	Mean	Max.	Min.	SD	Obs.
**BRI**					
REC	0.449357	3.310323	−3.948913	1.129932	1002
GDP	3.95578	49.48028	−38.41823	4.460221	1002
FDI	9.207394	11.46379	5.997877	0.834617	1002
TO	1.934765	2.640806	1.403227	0.212714	1002
**CEE**					
REC	0.520552	2.295484	−1.744727	0.765510	344
GDP	4.153678	12.99696	−14.46433	3.678390	344
FDI	9.218194	10.96456	7.525536	0.662621	344
TO	2.005775	2.280347	1.665460	0.138831	344
**CWA**					
REC	0.272340	1.814461	−3.948913	1.246526	194
GDP	15.31567	33.03049	−13.51939	5.188496	194
FDI	8.883112	10.23606	6.668569	0.687224	194
TO	1.891455	2.243909	1.465268	0.154345	194
**MEA**					
REC	−0.285949	2.114630	−3.00	1.309214	170
GDP	1.143672	49.48028	−38.41823	5.996282	170
FDI	9.376639	10.34335	7.812202	0.576680	170
TO	1.905971	2.283013	1.480676	0.180914	170
**SA**					
REC	0.937635	2.459650	−0.326979	0.739914	112
GDP	4.314932	17.03122	−2.243643	2.526166	112
FDI	8.701855	10.70424	5.997877	1.145838	112
TO	1.677881	2.066511	1.403227	1.145838	112
**SEA**					
REC	0.887780	3.310323	−2.6989891	1.212902	181
GDP	4.554989	13.63582	−3.784520	2.877514	181
FDI	9.692274	11.46379	6.648487	0.932703	181
TO	2.035158	2.640806	1.554975	0.271759	181

Max. denotes maximum, Min. represents minimum, and SD signifies Standard Deviation.

The highest renewable electricity consumption (2212.54) is recorded in China, while Turkmenistan had the lowest renewable electricity consumption (0.0003) in 2019. China and Lebanon have the greatest (2.116522893) and lowest (−21.11600443) GDP per capita values. Singapore (320.5635138) is at the top in globalization or trade openness.

### 4.1 Cross-sectional dependence test

This research started with the baseline model presented in Table 2 and used the Breusch-Pagan LM, Pesaran scaled LM, and Pesaran CD tests to examine the cross-sectional dependency of variables. As the probability is < 0.5, the null hypothesis is rejected as H0: Variables do not have a serial correlation.

**Table:** Serial Correlation Test

	BRI		CEE		MEA	
Test	Stat.	Prob.	Stat.	Prob.	Stat.	Prob.
**Breusch-Pagan LM**	5525.849	0.0000	958.8660	0.0000	241.1607	0.0000
**Pesaran scaled LM**	67.35643	0.0000	46.06329	0.0000	11.13065	0.0000
**Pesaran CD**	28.60598	0.0000	11.75485	0.0000	0.531051	0.5954
	**CWA**		**SA**		**SEA**	
***Test***	***Stat.***	***Prob.***	***Stat.***	***Prob.***	***Stat.***	***Prob.***
**Breusch-Pagan LM**	114.5133	0.0000	36.20925	0.0016	147.7128	0.0000
**Pesaran scaled LM**	7.327347	0.0000	3.872262	0.0001	10.82688	0.0000
**Pesaran CD**	7.226900	0.0000	−1.048614	0.2944	1.351272	0.1766

**Table 2:** Results of the base line model.

	BRI	CEE	MEA	CWA	SEA	SA
Vr,	Coef.	Coef.	Coef.	Coef.	Coef.	Coef.
**GDP**	0.029213***	−0.025281***	0.029765**	−0.266913***	0.073525***	−0.043110***
**TO**	−1.787053***	−2.494992***	−4.734048***	−1.692128	−1.833426***	0.886352**
**FDI**	0.0434624***	0.509256***	0.356110***	−1.041917**	0.684812***	0.460601***
**C**	−0.182807*	0.935531*	5.363849***	20.68204***	−2.353198***	−4.371623***

***Indicates significant at the 1%, ** Indicates significant at the 5%, and * significant at the 10% level is shown.

### 4.2 Second generation unit root test

Due to serial correlation, four tests are utilized in this work to establish the sequence of data integration: Levin and Lin, Breitung, Im Pesaran and Shin [[79], [80], [81]], and Fisher-PP. Table 3 shows the results of second-panel unit root testing. According to the results, the dataset has a unit root at level and is stationary at the first difference.

**Table 3:** Unit Root tests.

*Panel Countries*								
*Vr.*	*LLC*		*Breitung t-stat*		*CIPS*		*PP*	
	*I(0)*	*I (1)*	*I(0)*	*I (1)*	*I(0)*	*I (1)*	*I(0)*	*I (1)*
REC	7.35005	−25.7320***	6.70057	−4.78050***	1.61639	−21.3635***	208.887***	707.741***
FDI	−7.51391***	−27.6740***	−5.11549	54.0646***	−5.50169***	−22.0800***	236.516***	793.641***
GDP	−6.34976***	−25.9833***	25.5310***	79.6758***	−8.7952***	−14.2503***	14.1901***	36.8232***
TO	−4.38210***	−23.5759***	3.00155	−6.75865***	0.07688	−17.0719***	126.207	548.670***
CEE								
REC	0.5419	−17.8429***	−1.59897*	−11.2967***	−4.29042***	−13.9482***	130.258***	302.728***
FDI	−3.03571**	−14.9550***	−2.87200***	−11.5634***	−0.70655	−11.0908***	42.6851	260.121***
GDP	−4.91278***	−15.3982***	−0.10399	−3.30495***	−4.82614***	−13.1458***	79.1274***	258.811***
TO	−0.45383	−11.2877***	1.29920	−5.59112***	0.60163	−8.28521***	29.5718	170.773***
CWA								
REC	−1.34980	−8.25570***	1.18890	−0.04352**	−0.88844	−5.96153***	24.1126	84.7336***
FDI	−5.05871***	−10.1036***	1.73946	−1.77687***	−1.42297	−9.52266***	48.5693***	142.968***
GDP	−3.63601***	−9.94922***	0.52435	−3.62608***	−2.37424***	−8.10911***	42.4191***	146.921***
TO	1.35600	−4.67992**	1.47398	2.9546***	1.35077	−4.50008***	13.6962	68.9972***
SA								
REC	−0.65149	−6.15642***	0.30283	−3.57155***	−0.60384	−4.25094***	33.0948***	66.9894***
FDI	−3.16391***	−4.41925***	−1.16153	−1.94166***	−1.13094	−4.25297***	12.9709	65.9277***
GDP	2.88555	−0.96573***	5.11943	−3.62608***	0.20182	−2.35665***	10.8132	68.7099***
TO	−1.31575	−3.34523**	1.38599	−0.57368*	0.15726	−3.03637***	5.84415	35.9819***
SEA								
REC	0.26981	−2.28544***	−0.47317	−3.44042***	0.15637	−4.03232***	44.2327**	139.482***
FDI	0.21111	−8.17921***	−1.65439*	−2.10282***	−0.29460	−5.92186***	70.0549***	173.879***
GDP	4.14339	−3.17758***	7.41968	−5.80895***	1.14632	−3.23921***	34.0921**	130.905***
TO	−1.64964*	−36.2454**	−0.54685	−1.77150***	−1.59979*	−13.4573***	39.8445**	120.862***
MEA								
REC	−1.32555	−4.89689***	−0.86899	−3.77340***	0.37519	−5.16331***	19.8912	119.682***
FDI	−0.92904	−4.51324***	0.36787	−0.30826**	−2.58278**	−2.83215***	50.8896***	174.409***
GDP	−2.00870*	5.97386***	0.11962	−1.41952***	−2.41505***	−6.40417***	70.2850***	188.608***
TO	−0.80734	−5.72091**	0.81327	−2.94732***	−0.28808	−6.15373***	36.9803	151.133***

*** Indicates significant at the 1%, ** Indicates significant at the 5%, and * significant at the 10% level is shown.

### 4.3 Co-integration checks

To analyze the co-integration of variables, this study used two co-integration tests developed by Pedroni [84,85] and Kao [86]. The results of the co-integration tests are summarized in Table 4. This study observed three homogenous statistics and two heterogenous statistics of Pedroni [86,87] are statistically significant at 1% in BRI countries and its regions; this demonstrates that the alternative hypothesis about the existence of co-integration is accepted.

**Table 4:** Panel Co-integration tests.

	BRI		*CEE*		*MEA*	
Residual co-integration test by Pedroni [86,87]						
	*Stat.*	*Pr*	*Stat.*	*Pr*	*Stat.*	*Pr*
**Panel v-Statistics**	61.85165	0.0000	1.230628	0.1092	2.847310	0.0022
**Panel rho-Statistics**	1.853247	0.1681	2.692238	0.9965	1.894370	0.9709
**Panel pp-Statistics**	−9.252117	0.0000	−1.793846	0.0364	−2.786756	0.0027
**Panel ADF-Statistics**	−5.013338	0.0000	−4.677310	0.0000	−0.800251	0.0231
**Group rho-Statistics**	6.411627	0.1289	3.584839	0.9998	3.295827	0.9995
**Group PP-Statistics**	−8.091501	0.0000	−6.939207	0.0000	−3.530917	0.0002
**Group ADF-Statistics**	−1.147566	0.1256	−4.392661	0.0000	−1.424954	0.0771
**Kao** [88] **Cointegration Test**						
**ADF**	7.17.598	0.0000	−2.889336	0.0019	−2.973762	0.0035
	**CWA**		**SA**		**SEA**	
Residual co-integration test by Pedroni [86,87]						
	***Stat.***	***Prob.***	***Stat.***	***Prob.***	***Stat.***	***Prob.***
**Panel v-Statistics**	4.825849	0.0000	0.014995	0.4940	26.67107	0.0000
**Panel rho-Statistics**	0.763347	0.7774	−0.251648	0.4007	0.774140	0.7806
**Panel pp-Statistics**	−3.533649	0.0002	−4.336262	0.0000	−3.986463	0.0000
**Panel ADF-Statistics**	−3.102329	0.0010	−1.0406144	0.1343	−2.075264	0.0190
**Group rho-Statistics**	2.450229	0.9929	1.447830	0.9262	1.841955	0.9673
**Group PP-Statistics**	−2.945187	0.0016	−2.986617	0.0014	−3.509698	0.0002
**Group ADF-Statistics**	−2.604491	0.0046	0.153422	0.5610	−0.854375	0.1964
**Kao** [88] **Cointegration Test**						
**ADF**	−2.399751	0.0082	−1.731595	0.0417	2.038583	0.0207

Furthermore, the co-integration test statistics provided by Kao [88] demonstrated that the null hypothesis was rejected and accepted alternative hypothesis of cointegration. As a result, both co-integration checks disclose that the globalization index's renewable power consumption, economic growth, trade openness, and FDI are co-integrated and have a long-term connection.

### 4.4 PMG ARDL estimates

This study may now examine the short and long-term associations because co-integration among the variables has been proven. For this objective, the panel PMG ARDL approach is initially used in this work. Table 5, Table 6 show the predicted outcomes of the panel PMG approach. The short-run results of Eq. (9) are reported in Table 5. Based on the optimal structure, lag 1 is used. The acquired results reported that FDI is negative and insignificant with respect to renewable electricity consumption in BRI, CWA, MEA, and SEA countries. The 1% increase in FDI reduces 0.01% of renewable electricity consumption. Prior studies [25,31,59,86] have found a significant effect on energy use. The energy-conserving techniques brought by FDI also reduced renewable electricity consumption [66,[90], [91], [92], [93]]. Renewable electricity consumption might be higher during the planning phase [94]. Su et al. [95] reported that weak absorptive capacities could prevent firms from adopting energy-efficient technology.

**Table 5:** Short run estimates.

*REC is the dependent variable*						
	*BRI*		*CEE*		*CWA*	
*Vr.*	*Coef.*	*z- value*	*Coef.*	*z- value*	*Coef.*	*z- value*
FDI	−1.03	−0.54	0.00024	1.86*	−0.00007	−4.66***
GDP	−3.916556	−2.28**	−3.480441	−1.98**	−3.250823	−1.73**
TO	1.530833	1.03*	5.805423	4.08***	2.354455	2.63***
ECT	−0.0821197	−5.84***	−0.1395717	−4.42***	−0.1496543	−4.66*
	**MEA**		**SA**		**SEA**	
FDI	−0.00032	−1.33	0.00843	1.40	−0.000848	−1.12
GDP	−0.0426269	−0.01	0.9723277	2.07**	0.1237094	0.09*
TO	−3.201837	−0.51	0.6004809	1.23	−0.5482837	0.60
ECT	−0.0319218	−1.62*	−0.0173745	−0.64*	−0.0066962	−1.02*

*** Indicates significant at the 1%, ** Indicates significant at the 5%, and * significant at the 10% level is shown.

**Table 6:** Long run estimates.

*REC is the dependent variable*						
	*BRI*		*CEE*		*CWA*	
*Vr.*	*Coeff.*	*Z- value*	*Coeff.*	*Z- value*	*Coeff.*	*Z- value*
FDI	0.00046	5.05***	0.000469	4.53***	−0.00067	4.54***
GDP	69.4072	9.32***	70.49162	8.27***	37.2763	3.88***
TO	9.337392	9.35***	7.38365	5.96***	0.2729975	0.12
	**MEA**		**SA**		**SEA**	
FDI	−0.000156	−1.67*	0.000183	2.44**	−0.000186	1.03
GDP	−217.8512	−2.23**	−6.616565	−0.46	−6.296859	0.09*
TO	144.7503	3.17***	−0.0926828	−0.03	289.1478	1.10

*** Indicates significant at the 1%, ** Indicates significant at the 5%, and * significant at the 10% level is shown.

Economic growth in the short run was negatively correlated with renewable electricity consumption in BRI, CWE, CEE, and MEA countries. The result showed that a 1% escalation in growth lowers renewable electricity depletion by 0.039%, 0.034%, 0.0325%, and 0.00042% respectively. It can be explained that as income per capita increases, the capacity to purchase durable technology with efficient energy consumption increases, due to which renewable electricity consumption decreases. Trade openness is positively correlated with renewable electricity consumption in BRI, CWA, CEE, and SA countries. A 1% upsurge in globalization enhanced renewable electricity use by 0.015% in BRI countries. Negative ECT verified the association between trade openness, FDI, economic growth, and renewable electricity consumption for all regions in the short-run. ECT in BRI countries also revealed that an 8% decrease in renewable electricity consumption is rectified in the near term by deviations from long-run equilibria.

Table 6 shows the long-run elasticities. For BRI, CEE, and SA economies, there is a positive and strong relationship between FDI and renewable electricity use. For BRI countries, empirical results revealed that an increase in FDI upsurges 0.04% renewable electricity use while holding other things constant; this finding is consistent with the findings of Phrakhruopatnontakitti et al. [71]. When investment increases in the economy, energy consumption also increases, affecting renewable electricity consumption. Moreover, governments of different countries struggle with the diffusion of old technology with new energy-efficient technology.

By holding all other variables fixed, a 1% rise in economic growth is associated with a 0.69% increase in renewable electricity usage. These optimistic associations in BRI, CEE, and CWA countries are consistent with the results reported previously [96,97]. During 1980 and 1990, smaller unity coefficients indicated energy inefficiency in the labor-intensive industry [30,95]. Moreover, it also provides indirect proof for the growth decomposition effect [98,99]. If the composition effect surpasses the scale effect, we consider its efficiency in renewable electricity utilization, but in this study the composition effect falls belowunity, sp we believe it is inefficient utilization of energy [100].

In the long term, there is a significant positive association among renewable electricity usage and trade openness in BRI, CEE, CWA, MEA, and SEA countries. The outcome is in line with Shahbaz [62], Ang [105] and Jalil [106]. Table 5, Table 6 suggest that the difference in results is due to different policies and economic situations in the region. Hence, ECT in all panel countries is negative and significant, showing the model's accuracy.

### 4.5 DH panel causality test

The DH panel causality test results shown in Table 7 revealed that trade openness and renewable electricity consumption have two-way causality in BRI and MEA countries and unidirectional causality in CEE, CWA, SA, and SEA countries. Economic development and renewable electricity intake have unidirectional causality among SEA and SA nations, while in MEA countries have bidirectional causality. Results also reveal unidirectional causality among FDI and renewable electricity consumption in BRI, CEE, MEA, and SA countries.

**Table 7:** Panel causality test.

BRI		CEE	
Null Hypothesis	F-stats.	Null Hypothesis	F-stats.
FDI ⟹ REC	3.59643**, 0.86752	FDI ⟹ REC	3.07882**, 1.27086
TO ⇔ REC	2.99405**, 1.72601*	TO ⟸ REC	0.14244, 6.53485***
TO ⇔ FDI	2.52607**, 3.34229**	TO ⟹ FDI	2.31865*, 0.48056
GDP ⇔ FDI	2.53076***, 6.10853***	GDP ⇔ FDI	5.48939***, 7.62580***
GDP ⇔ TO	9.39103***, 1.97697*	GDP ⟹ TO	17.6537**, 1.37246
**SA**		**MEA**	
FDI ⟹ REC	4.76965**, 3.76334	FDI ⟹ REC	3.89311**, 1.47166
TO ⟹ REC	4.64206**, 1.62144	TO ⇔ REC	1.75266*, 1.91873*
GDP ⟸ REC	1.83217, 6.95805***	GDP ⇔ REC	6.10769***, 2.46567**
TO ⟹ FDI	4.21653*, 3.14281	TO ⇔ FDI	3.25207**, 2.10426*
GDP ⟹ FDI	2.78363***, 0.79962	GDP ⟸ FDI	0.69798, 1.67518*
FDI ⟹ REC	4.76965**, 3.76334	GDP ⇔ TO	3.09386**, 6.00802***
**CWE**		**SEA**	
TO ⟸ FDI	0.42899, 2.66318**	GDP ⟹ TO	1.76304*, 0.45094
TO ⟹ REC	4.36363***, 0.58681	TO ⟸ REC	0.52182, 2.03634*
GDP ⟸ FDI	0.51764, 0.69325*	GDP ⟹ REC	3.38198**, 0.28384

***Indicates significant at the 1%, ** Indicates significant at the 5%, * significant at the 10% level is shown, ⇔ indicates bidirectional causality, and ⟹ indicates unidirectional causality.

## 5 Discussion

This is essential to determining if the variables in this study are stationary before applying the panel cointegration test since nonstationary data frequently provides erroneous regression findings. The same autoregressive parameter assumption and the different autoregressive parameter assumption tests are two of the four primary types of panel unit root tests. In this study, four tests are conducted: the Levin-Lin-Chu (LLC), Breitung t-stat, Im-Pesaran-Shin (IPS), and Phillip Perron (PP) tests. The LLC test is a widely used method for testing the same assumption about an autoregressive parameter, while the IPS test is a well-liked method of testing an assumption about an alternative assumption about an autoregressive parameter. It is apparent that although all variables are stationary at the initial difference I(1), the majority of data series are nonstationary at level I(0). As a result, all of the variables included in this study are stationary at the point of first difference I(1). The results of the Pedroni and kao co-integration tests are statistically significant at 1% in BRI countries and their regions. As a result, both regionally and across panels, the findings of the panel cointegration tests support the existence of cointegration links among the consumption of renewable electricity, trade openness, foreign direct investment, and economic growth. Panel PMG ARDL model represents short and long run results for BRI nations and their regions. When compared to long-term connections, short-term relationships between variables are distinct. In CEE, SA, and CWA countries, as well as the BRI nations as a whole, trade openness has positive and negative effects on the use of renewable power, but it has negative consequences in MEA and SEA countries. The consumption of renewable power per capita will rise in the short term in BRI countries by 1.53%, 5.80% in CEE countries, 2.35% in CWA countries, and 0.60% in SA countries if trade openness grows by 1%. Though few studies have focused on the connection between renewable energy and trade openness, the majority of them indicated that trade openness had a favorable influence on the use of renewable energy [62, 105, 106], which was consistent with our findings. The empirical findings of Alam et al. [61] and Azam et al. [63], who conducted research in Turkey and the OECD nations, respectively, are comparable to the empirical results of the short-run negative association between economic growth and renewable electricity use. Additionally, the short-term data indicate that in the BRI, MEA, and SEA nations, foreign direct investment has a negative influence on the use of renewable power [66,[90], [91], [92], [93]].

In terms of the long-term connection, the findings show that economic growth and trade openness greatly increase the consumption of renewable power in BRI and CEE countries, but trade openness has the opposite impact in SA countries. The findings imply that economic growth, foreign direct investment, and trade openness increase the long-term usage of renewable power in BRI nations. The undeveloped economic sector in MEA, SA, and SEA nations might be the cause of the inverse relationship between economic growth and renewable electricity use. For instance, low-quality entities and high production costs discourage investment in the infrastructure and technology of renewable energy [31,35]. A major barrier to the expansion of renewable energy in BRI has been the ineffective management of trade openness and foreign investments [44], and financial markets are ineffective in fostering the sector's development. The BRI nations' collective usage of renewable power is positively impacted by economic growth. This is because outdated technology is being used, which uses more power. On the one hand, more energy, particularly renewable energy, will be consumed once a nation or area has achieved economic growth in order to maintain the rate of expansion. While on the other side, economic expansion may lead to the development of new renewable energy technologies that will help the industry thrive.

## 6 Conclusion and policy implications

The above study looked at the strong connection among globalization, economic evolution and renewable electricity consumption using the PMG ARDL approach in BRI countries and their regions during 2000–2020. Co-integration tests revealed long-run associations among selected variables. In the long term, there is a positive association among FDI and renewable power consumption, while in the short run, there is a negative relationship for BRI nations. Such findings might be interpreted as FDI between nations increasing renewable power consumption and improving it in the long run, but decreasing it in the near term owing to technological advancement. Economic development is positively related to renewable electricity usage in the long term and negatively related in the short term. Furthermore, In both the long and short run, trade openness is positively related. The results showed an increase in trade demands for higher production of electricity.

The empirical findings of this study revealed a number of policy implications. Initially, it is essential to remember that attracting globalization increases the industrial sector's constant upgrading and modifies the economy's structure, which can be helpful to curtail energy consumption and expand environmental quality. Moreover, mutual efforts should be enhanced between domestic industries and globalization related to energy. These findings recommend that globalization improves energy/electricity efficiency and productivity and enhances the economies' energy/electricity conservation concepts. The increase in globalization and economic growth significantly increases the renewable electricity consumption in BRI countries and their regions. In conclusion, we proposed that these countries adopt sustainable energy-efficient technology instead of an erratic one, and the government must introduce the programs to enhance the absorptive capacity of energy-intensive local firms. Investments in renewable energy projects should be encouraged while using both equity and debt financing. A crucial method of luring financial resources into the renewable energy sector is through international finance.

This study has significant limitations, such as the fact that further research may be done on the consumption of power at the corporate level and on various sources of renewable energy.

## Author contribution statement

Gulzara Tariq: Conceived and design the analysis; Wrote the paper.

Huaping Sun: Analyze and interpret the data.

Unai Fernandez-Gamiz: Analyze and interpret the data.

Sofia Mansoor: Contributed analysis of data.

Amjad Ali Pasha: Contributed analysis of data.

Sajjad Ali: Contributed analysis of data.

Muhammad Sohail Khan: Analyze and interpret the data.

## Funding statement

Huaping SUN was supported by the 10.13039/501100012456National Social Science Fund of China [21AZD067], and the 10.13039/501100001809National Natural Science Foundation of China (72243005).

Unai Fernandez-Gamiz was supported by the government of the Basque Country [ELKARTEK21/10 KK-2021/00014 & ELKARTEK22/85 KK-2022/00043].

## Data availability statement

Data will be made available on request.

## Declaration of interest’s statement

The authors declare no competing interests.

## Abbreviations

BRI: Belt and Road Initiative
PMG: Pooled Mean Group
ARDL: Autoregressive distributed lag
FDI: Foreign direct investment
GDP: Gross domestic product
REC: Renewable electricity consumption
TO: Trade openness
LLC: Levin, Lin and Chu
ADF: Augmented dickey-fuller
PP: Phillip perron
CEE: Central and eastern europe
CWA: Central and western asia
SA: South asia
SEA: South-east asia
MEA: Middle east and africa
LPS: Lm Pesaran and Shin
